# Mutations in the KDM5C ARID Domain and Their Plausible Association with Syndromic Claes-Jensen-Type Disease

**DOI:** 10.3390/ijms161126022

**Published:** 2015-11-13

**Authors:** Yunhui Peng, Jimmy Suryadi, Ye Yang, Tugba G. Kucukkal, Weiguo Cao, Emil Alexov

**Affiliations:** 1 Computational Biophysics and Bioinformatics, Department of Physics, Clemson University, Clemson, SC 29634, USA; yunhuip@clemson.edu (Y.P.); tugbak@g.clemson.edu (T.G.K.); 2 Department of Genetics and Biochemistry, Clemson University, Clemson, SC 29634, USA; jsuryad@clemson.edu (J.S.); yyang9@clemson.edu (Y.Y.)

**Keywords:** X-linked syndromic Claes-Jensen type disease, sequence variants, folding free energy changes, binding free energy changes, molecular dynamics, free energy perturbation

## Abstract

Mutations in *KDM5C* gene are linked to X-linked mental retardation, the syndromic Claes-Jensen-type disease. This study focuses on non-synonymous mutations in the KDM5C ARID domain and evaluates the effects of two disease-associated missense mutations (A77T and D87G) and three not-yet-classified missense mutations (R108W, N142S, and R179H). We predict the ARID domain’s folding and binding free energy changes due to mutations, and also study the effects of mutations on protein dynamics. Our computational results indicate that A77T and D87G mutants have minimal effect on the KDM5C ARID domain stability and DNA binding. In parallel, the change in the free energy unfolding caused by the mutants A77T and D87G were experimentally measured by urea-induced unfolding experiments and were shown to be similar to the *in silico* predictions. The evolutionary conservation analysis shows that the disease-associated mutations are located in a highly-conserved part of the ARID structure (N-terminal domain), indicating their importance for the KDM5C function. N-terminal residues’ high conservation suggests that either the ARID domain utilizes the N-terminal to interact with other KDM5C domains or the N-terminal is involved in some yet unknown function. The analysis indicates that, among the non-classified mutations, R108W is possibly a disease-associated mutation, while N142S and R179H are probably harmless.

## 1. Introduction

Epigenetic processes regulate gene expression and are essential for development and differentiation of cells [1]. Histone proteins are the major components of chromatin, acting as spools around which DNA winds. Particularly, histone lysine methylation is an important epigenetic process which regulates chromatin structure and gene transcription [2,3]. Due to this, loss of balance of histone lysine methylation has been found to have a profound effect on the diverse biological processes and to be involved in many diseases, including cancer development [4,5,6].

**Figure 1:**
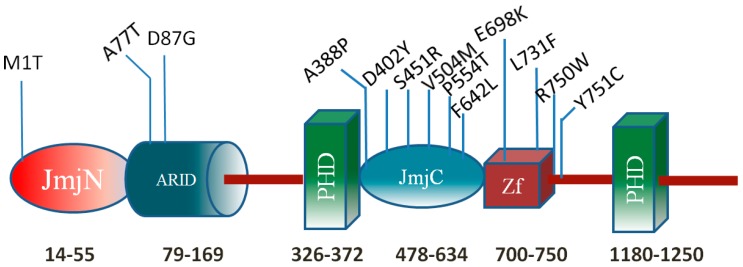
KDM5C protein domains. The numbers indicate approximate domain boundaries. The known disease-associated missense mutations are provided as well.

This work focuses on a particular histone protein, the KDM5C protein of 1560 aa, which is a member of the SMCY homolog family. The KDM5C protein specifically reverses tri- and di-methylation of Lys4 of histone H3 (H3K4), helps maintain the dynamic balance of histone H3K4 methylation states, and also plays a crucial role in functional discrimination between enhancers and core promoters [7,8,9]. It is a multi-functional protein, which contains highly-conserved domains, including ARID/Bright, JmjN, JmjC, C5HC2 zinc finger, and two PHD zinc finger domains (Figure 1). These domains were shown to have specific functions alone or to function in concert with the other KDM5C domains. Thus, the ARID (A–T rich interaction domain) is a helix–turn–helix motif-based DNA-binding domain, which is highly conserved in all eukaryotic proteins and plays important roles in development, tissue-specific gene expression, and cell growth regulation [10,11]. The DNA sequence binding preference is still unclear for the ARID domain of KDM5C. The other domain, JmjC, catalyzes demethylation of H3K4me3 to H3K4me1 [7]. The JmjN domain and its interaction with the JmjC catalytic domain are important for the KDM5C function [12]. The N-terminal PHD zinc finger is a histone methyl-lysine binding motif and was shown to have a preferential binding to histone H3K9me3 [7,13].

Previous studies have shown that many mutations in the *KDM5C* gene cause X-linked mental retardation (XLMR), the syndromic Claes-Jensen-type disease [7,14,15]. Mental retardation (MR) generally causes significant limitations both in intellectual functioning and in adaptive behavior, covering the social and practical skills that originate before the age of 18 years [16]. The estimated prevalence of MR among the general population is around 1%–3% [15,17]. The frequency of mutations in the *KDM5C* gene approximately accounts for 2.8% to 3.3% of families with XLMR [18]. Thirteen missense mutations associated with XLMR, the syndromic Claes-Jensen-type disease in *KDM5C* have been reported to date and affected individuals with *KDM5C* mutations show a mild-to-severe range of intellectual disability. Most of mutations are located in JmjC domain, ZF domain (C5HC2 zinc finger domain), and inter-domain regions and affect the demethylation activity [7,8]. The severity of associated XLMR is roughly related to the cellular demethylase activities of KDM5C mutants [19]. In this study, we focus on the mutations in the ARID domain. Two MR associated mutations (A77T and D87G) are reported in the ARID domain [14,20]. The D87G mutation causes mild to moderate MR including aggressive behavior, epileptic seizures, and speech impairment, while the A77T results in severe MR including speech impairment, short stature, seizures, microcephaly, hyper reflexia, and aggressive behavior [14,20]. However, recent work has shown that the D87G has a minimal effect on KDM5C demethylase activity *in vivo* [19] indicating that the disease-associated effect is not demethylation. Combined with the lack of data for the molecular effect of A77T mutation, it can be concluded that the disease-associated effects of both A77T and D87G mutations are unknown. In this work, we extend the list of mutations, which will be investigated, to include three currently non-classified missense mutations in the ARID domain. The non-classified mutations are R108W(rs146232504), N142S(rs377166019), and R178H(rs201805773), taken from the NCBI dbSNP database [21]. They were identified from population cohorts participating in the NHLBI Exome Sequencing Project [22]. This project is designed to identify genetic variants in coding regions of the human genome that are associated with heart, lung, and blood diseases, and the group included 200,000 individuals. However, there is no data about the linkage of these mutations with a particular disease. This motivates us to investigate the molecular mechanism of all abovementioned mutations, disease-associated and non-classified, and to infer plausible XLMR linkages with some of the non-classified mutations. The allele frequency of the mutations R108W, N142S, R179H are 0.00001151, 0.00001159, and 0.0002497 taken from the ExAC database [23]. The frequency of the other two mutations is not currently available in the database.

Disease-associated mutations are often found to alter protein structure, dynamics and interaction, and cause deficiency of important protein functions [24,25,26,27,28]. Investigating mutations’ effects is important for understanding the molecular mechanisms of disease-associated mutations and discriminating disease-causing and harmless mutations. Protein stability and protein interactions can be quantified by folding free energy change (∆∆G) and binding free energy change (∆∆∆G). In this study, we analyze the effects of diseasing-associated and currently non-classified mutations on ARID domain stability and ARID-DNA binding affinity utilizing webservers, third-party software, molecular dynamics (MD) and free energy perturbation (FEP) methods. Additionally, our free energy calculations results are further validated by experiments. Urea-induced unfolding monitored by circular dichroism spectroscopy is used to determine the unfolding free energy of the wild-type ARID domain, and the two disease-associated mutants A77T and D87G.

## 2. Results

### 2.1. Protein Stability Changes due to Mutations

We applied the free energy perturbation theory (FEP) to analyze two disease associated (A77T, D87G), and three non-classified (R108W, N142S, R179H), mutations. The calculated folding and binding free energy changes caused by mutations are shown in Table 1. It can be seen that the energy changes are predicted to be relatively small, being less than 1 kcal/mol in the majority of cases, with the notable exception of FEP calculated folding free energy changes involving Arg residue. A similar effect of over-predicting the magnitude of the change of the folding free energy involving the Arg group was noticed in another study [29]. Further investigations are needed to reveal the source of the over-estimation of the changes caused by Arg mutants, but for completeness, these calculated energies will be used as they are in the present study. The average folding free energy changes predicted by webservers and third-party software are all relatively small, being less than 1 kcal/mol. The FEP calculated binding free energy changes indicate that mutations R108W and R179H cause relatively large changes compared to other mutations.

**Table 1:** The calculated binding and folding free energy changes due to mutations in kcal/mol. ∆∆G > 0 indicates stabilization, while ∆∆G < 0 shows destabilization. The “Folding (average)” column shows the average folding free energy changes calculated using the average folding free energy changes predicted by FEP, webservers, and third party software (left), and folding free energy changes predicted by webservers and third party software (right). The changes of the binding free energy were obtained only with FEP, since no reliable third-party tool currently exist.

Mutation	NeEMO (Folding)	PopMusic (Folding)	I-Mutant (Folding)	DUET (Folding)	CUPSAT (Folding)	Foldx (Folding)	FEP (Folding)	Folding (Average)	FEP (Binding)
A77T	−1.03	−0.22	−0.75	−0.76	−0.29	−1.40	0.13	−0.74/−0.62	−0.35
D87G	−0.16	−0.49	−0.47	−0.729	−0.16	−0.60	−0.28	−0.43/−0.41	0.73
R108W	−0.36	−0.86	−1.32	−0.26	0.28	−0.18	−11.29	−0.45/−1.99	−1.44
N142S	−0.21	−0.27	−0.09	−0.02	0.17	−0.3	−0.98	−0.12/−0.24	0.64
R179H	−0.71	0.06	−0.15	−0.72	0.28	0.746	−8.78	−0.08/−1.32	−3.06

As mentioned above, the mutations were predicted to have a small effect on both the folding and binding free energy (excluding the FEP results for Arg-involving mutations). This suggests that the disease-associated effect may not be related to these energies but may be linked to structural distortion or change of the internal dynamics/flexibility of the ARID domain caused by the mutations. Therefore, we review the structural features of the mutation sites below and elaborate on their possible linkage with the predicted effect of folding and binding free energy.

### 2.2. Effect of Mutations on Protein Structure 

**Figure 2:**
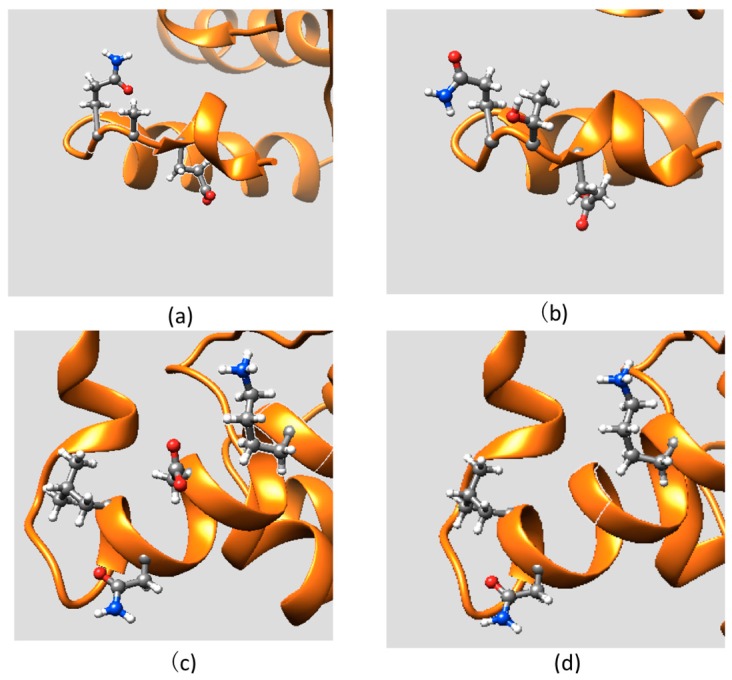
The side chain conformations of two disease-associated mutations mapped onto the KDM5C ARID domain: (**a**) part of the ARID domain zoomed at the WT position of A77; (**b**) part of thhe ARID domain zoomed at the MT position of T77; (**c**) part of the ARID domain zoomed at the WT position of D87; and (**d**) part of the ARID domain zoomed at the MT position of G87.

**Figure 3:**
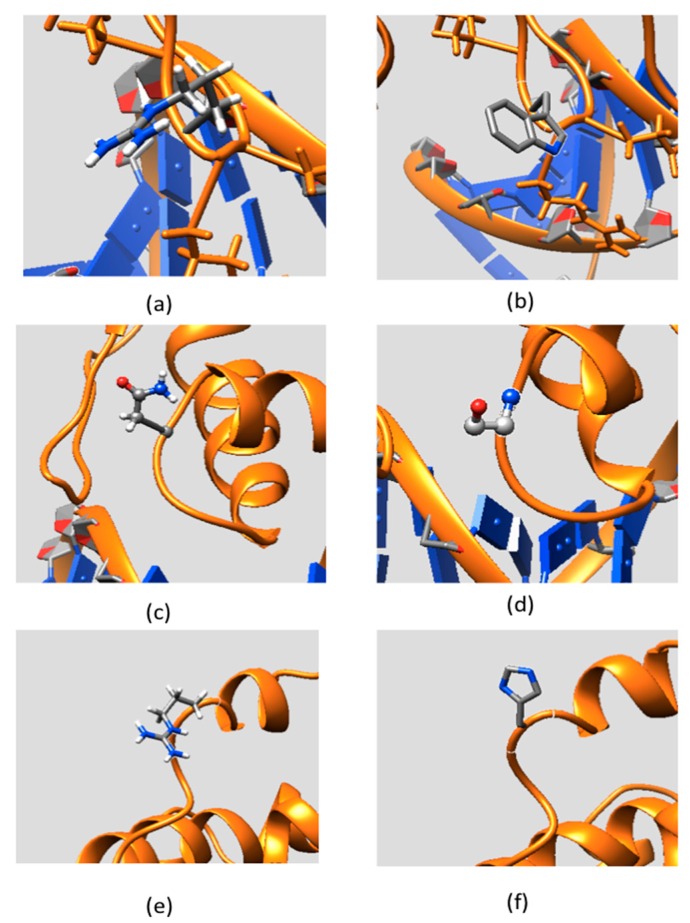
The side chain conformation of non-classified mutations mapped onto the KDM5C ARID domain: (**a**) part of the ARID domain zoomed at the WT position of Arg108; (**b**) part of the ARID domain zoomed at the MT position of Try108; (**c**) part of the ARID domain zoomed at the WT position of Asp142; (**d**) part of the ARID domain zoomed at the MT position of Ser142; (**e**) part of the ARID domain zoomed at the WT position of Arg179; and (**f**) part of the ARID domain zoomed at the MT position of His179.

To analyze the mutations’ plausible effect on the protein structure, here we investigated the side chains and backbone conformational changes resulting from mutations and discuss them with respect to structural integrity of the ARID domain and its interactions with DNA. The mutant is introduced into the structure using the Mutator Plugin, Version 1.3 in VMD [30]. After that, the mutant structures were subjected to 10,000 steps of energy minimization to relax the structure and remove possible conflicting contacts. The structures are then visualized in UCSF chimera [31]. The side chain conformation of the residues within 5 Å of the WT position or MT position, are shown in Figure 2 and Figure 3, respectively. Figure 2 shows side chain conformation of two disease-associated mutations mapped on the KDM5C ARID domain. The A77T mutation involves substitution of a hydrophobic Ala by a polar Thr and is located in a short turn of the ARID N-terminal. The mutation site is far away from the DNA binding interface and it is solvent-exposed. Neither the wild-type A77 nor the mutant T77 were found to be involved in any specific interactions (Figure 2a,b) The mutation D87G is located in Helix 1 of the ARID domain and a charged residue, Asp, is substituted by a small residue, Gly. This mutation site is also far away from the DNA binding interface and it is totally solvent-accessible. The wild-type residue, D87, is not involved in any specific interaction and its side chain faces the water (Figure 2c,d). Based on these structural observations and the results of folding free energy calculations, it can be summarized that these mutations do not solely affect the stability and the structure of the ARID domain. Similarly, since the mutation sites are far away from DNA, the binding interface, and the binding free energy is not predicted to be affected, one can assume that the mutations have minimal effect on ARID-DNA recognition.

**Figure 4:**
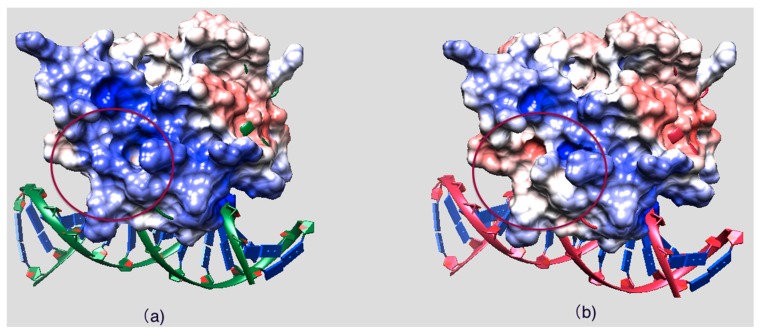
(**a**) Electrostatic potential of the WT KDM5C ARID domain; and (**b**) the electrostatic potential of the KDM5C ARID domain with mutation R108W. The mutation site is marked with a red circle. The positive potential region is colored with bule and the negative potential region is colored with red.

**Figure 5:**
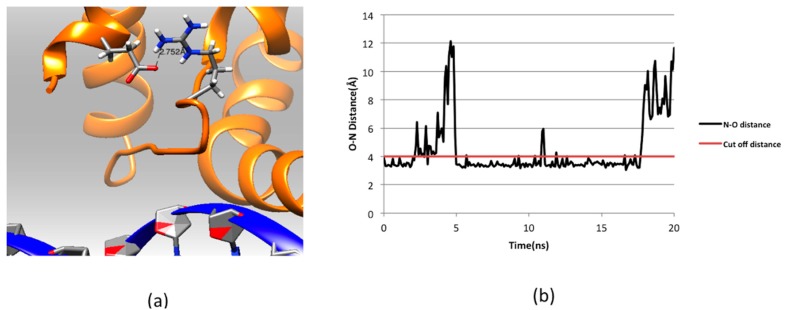
(**a**) Part of the ARID domain zoomed at the salt bridge Glu74-Arg108; and (**b**) salt bridge analysis for Arg108 and Glu74 in the KDM5C ARID domain: N–O distance shows the distance between oxygen atom of Glu74 and nitrogen atom of Arg108 in the 20 ns simulation. The cutoff distance of forming salt bridge is 4 Å and marked with red line in the graph.

Figure 3 shows the side chains and backbone conformations of non-classified mutations mapped onto the ARID domain. The R108W is a positively-charged residue, Arg, substituted by an uncharged hydrophobic residue, Trp. This mutation occurs in the loop between Helix 1 and Helix 2 and is located close to the DNA binding interface (Figure 3a,b). Since the mutation drastically changes the physico-chemical property of the wild-type residue, it can be anticipated that this mutation may cause significant conformational changes. To address this possibility, we performed 20 ns MD simulations of the ARID domain and DNA complex and it was found that R108 does not form a direct hydrogen bond with DNA. Thus, the wild-type residue, R108, is probably not involved in specific interactions with DNA but may provide long-range steering towards the negatively-charged DNA. Figure 4 shows the electrostatic potential of WT KDM5C ARID domain and the ARID doman with mutation R108W generated by DelPhi software [32,33,34]. It can be seen that the electrostatic potential at the mutation site is changed from positive to negative upon the mutation. Since the DNA is highly negatively-charged, this electrostatic potential change nearby the DNA binding interface will probably decrease the ARID–DNA binding affinity and specificity, which is consistent with predictions of the protein binding free energy changes. Further, salt bridge analysis indicated that the R108 forms a transient salt bridge with the neighboring amino acid, E74. Figure 5b shows the distance between the oxygen atom of E74 and the nitrogen atom of R108 in the MD simulation of the ARID domain and DNA complex. Using a cut-off distance of 4 Å as an indication of formation of a salt bridge, it was found that such a salt bridge is formed in 17.4 ns out of 20 ns (87% of the simulation time). Thus, the mutation R108W will delete the salt bridge and will probably affect the protein’s stability, which is consistent with prediction of the protein folding free energy changes. The other mutation, N142S, occurs in a loop between Helix5 and Helix6, and results in a polar uncharged residue, Asn, substituted by another polar uncharged, but smaller, residue, Ser (Figure 3c,d). Such a mutation preserves the biophysical characteristics of the mutation site and is expected not to affect the stability and structural integrity of the ARID domain. The mutation R179H involves a positively-charged residue, Arg, substituted by a polar residue, His. It is located in the loop of the ARID domain C-terminal, which is far from the DNA binding interface and is totally solvent-exposed (Figure 3e,f). 

### 2.3. Effect of Mutations on Protein Dynamics

Here, we investigate the ARID domain structural integrity through backbone Root Mean Square Deviation (RMSD) and Root Mean Square Fluctuation (RMSF) of the disease-associated mutations and non-classified mutations based on 100 ns MD simulations (Supplementary Material, Figures S2 and S3). The backbone RMSD is calculated for the whole ARID domain for both the wild-type and mutant proteins. The results indicate that all mutations show insignificant effects on the RMSD distribution of the whole ARID domain, which is consistent with our prediction of protein stability changes, excluding the R108W mutant. However, it is quite possible that the decrease in the folding free energy predicted for the R108W mutant may not be sufficient to cause large alterations of the conformational dynamics and the ARID domain can remain intact. 

### 2.4. Residue Conservation via Multiple Sequence Alignment

**Figure 6:**
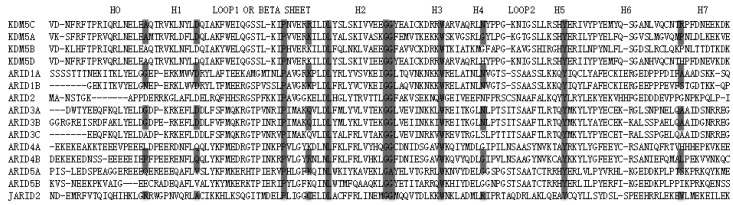
Sequence alignment of human ARID-containing proteins. The mutation sites considered in this study are marked with grey dash line. The six most highly conserved residues are marked with a grey solid line. The helices from H0 to H7, and loops, are labeled at the top of the figure. The sequences are aligned with T-Coffee [35]. Similar results were obtained using the Clustal Omega webserver.

Further, we investigate the conservation pattern of the KDM5C ARID domain amino acid positions based on the sequence alignment of human ARID domain proteins. The alignment (Figure 6) shows that the two disease-associated mutations (A77T and D87G) are conserved in the KDM5 family and D87 is conserved in ARID1, ARID2, and ARID3 families, as well. All non-classified mutations are not conserved in the alignment, including the alignment of only KDM5 family members. However, position 108 is predominantly taken by positively-charged residues, either Arg or Lys. Thus, a substitution to hydrophobic, uncharged Trp may not be tolerable. Combined with the predicted large change of the folding free energy and the change of the electrostatic potential, R108W mutation is predicted to be disease-associated. The other two non-classified mutations, N142S and R179H, occur at sites that are not conserved and there is no pattern to indicate the conservation of physico-chemical property of the wild-type residue. Even more, the substitutions Asn to Ser and Arg to His are found to exist in some family members (ARID3 and ARID4A), which suggest that such substitutions are tolerable.

Overall, the most highly conserved parts of the ARID domain are located on Loop1, Helix2, Helix3, Helix4, Loop2, and Helix5. Recent study showed that the KDM5A ARID domain binds DNA through the motif CCGCCC and the DNA binding interface includes Loop1 and a helix-turn-helix DNA binding motif formed by Helix4, Loop2, and Helix5 [36]. More specifically, six key residues (Pro103, Lys112, Gly123, Gly124, Trp134, and Tyr 157) are conserved in all human ARID-containing proteins, which indicates their importance for protein function. 

### 2.5. Evolutionary Conservation and Protein Interacting Investigation Using the ConSurf Server and IBIS Server

**Figure 7:**
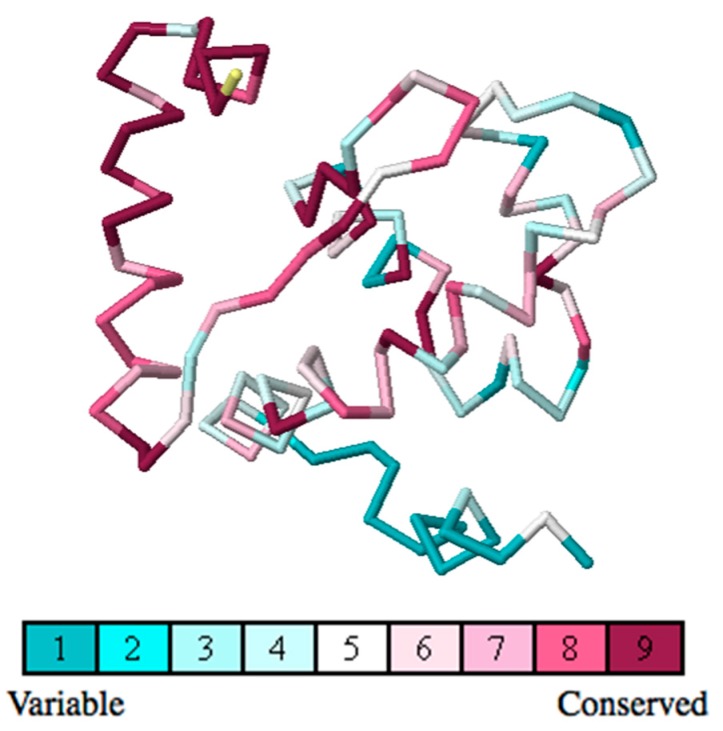
Evolutionary conservation analysis of the ARID domain using the ConSurf Server. The conservation grades are color-coded onto each amino acid of the KDM5C ARID domain.

The ConSurf server is a bioinformatics tool for estimating the evolutionary conservation of amino/nucleic acid positions in a protein/DNA/RNA molecule based on the phylogenetic relations between homologous sequences. The ConSurf server result (Figure 7) shows that the N-terminal of the ARID domain is one of the most highly-conserved parts in the ARID domain, which is probably essential for protein’s function. We also predict the protein interacting partners and binding sites in the KDM5C ARID domain using the NCBI Inferred Biomolecular Interactions Server(IBIS) [37]. The results show that Asp87 is a plausible zinc ion binding site. This binding sites is not verified experimentaly, but offer an implication that the N-terminal of ARID may be involved in some currently-unknown function.

### 2.6. Experimental Results

The mutations A77T and D87G affect the overall structure of the ARID domain slightly, but the percentage of each secondary structure of the mutants was in the same range as the wild-type (Table 2). In general, the effects of both A77T and D87G are the increase of the unordered structure percentage of the protein. While in the A77T mutation, the proportion of the structure shifted from alpha helix and turns to unordered; in D87G the shift came from of alpha helix and beta strand.

**Table 2:** Percentage of secondary structures of ARID proteins analyzed by using CONTINNLL [38] with the online tool Dichroweb [39].

Protein	Helix	Strand	Turns	Unordered
WT	14%	31%	20%	35%
A77T	13%	31%	19%	38%
D87G	13%	30%	20%	38%

**Figure 8:**
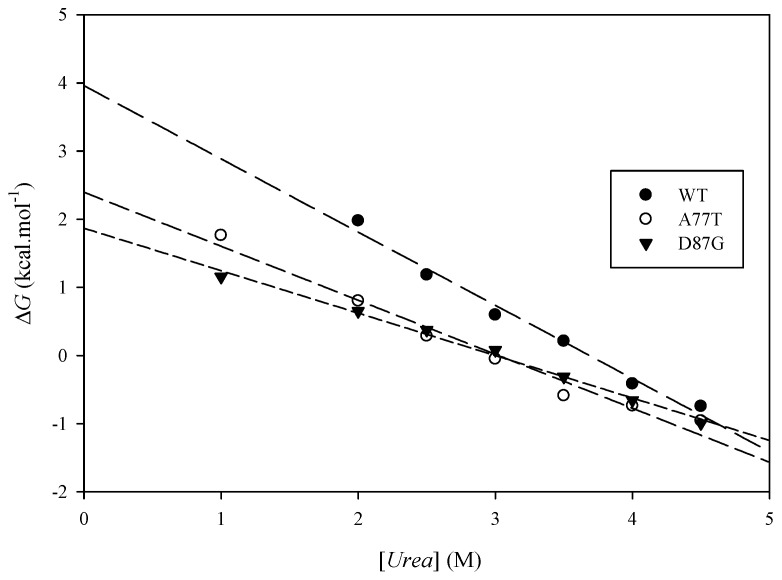
A representative plot of ∆*G* for ARID WT, A77T, and D87G unfolding as a function of urea concentration.

The results from the urea denaturation experiments (Table 3) indicate that both mutations caused a lower integrity protein structure (lower ∆G, easier to denature) than the wild-type, where the A77T is relatively more stable compared to D87G (but the difference is very small). There is a difference of the free energy of unfolding value of the ARID wild-type and the mutants (Figure 8). The two different methods to calculate the ∆∆G yields different value but the trends are the same, where the two mutants are less stable than the ARID wild-types, and D87G is less stable than A77T. The ∆∆G of the mutants A77T and D87G obtained by urea-induced unfolding monitored by CD are in the same order of magnitude compared to the *in silico* folding free energy predictions (Table 1).

**Table 3:** Results from an analysis of urea denaturation curves for ARID Wild-Type, A77T, and D87G variants.

Protein	$\Delta G_{app}^{H_{2}O}\;$(kcal·mol^−1^)	$\Delta \Delta G_{app,1}^{H_{2}O}\;$(kcal·mol^−1^)	Urea Concentration (M)	$\Delta \Delta G_{app,2}^{H_{2}O}\;$(kcal·mol^−1^)
ARID WT	3.51 ± 0.32		3.99 ± 0.02	
A77T	2.41 ± 0.05	1.10	3.07 ± 0.02	0.70
D87G	1.82 ± 0.01	1.70	2.99 ± 0.03	0.76

## 3. Methods and Experimental Section

### 3.1. Structures

**Figure 9:**
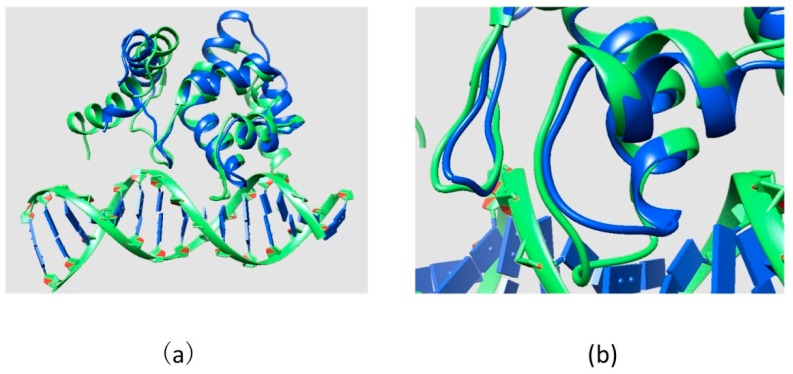
(**a**) Structural alignment between the KDM5C ARID domain and dead ringer ARID-DNA complex; and (**b**) part of structural alignment zoomed at DNA binding interface. Dead ringer ARID-DNA complex is marked with green and the KDM5C ARID domain is marked with blue.

The ARID domain contains 90 amino acids and its sequence is mapped onto the KDM5C protein sequence from position 79 to 169. There is an NMR structure of the KDM5C ARID domain (PDB ID: 2JRZ) [40] in the Protein Data Bank (PDB) [22], which was used for modeling the ARID domain stability. The modeling of the effect of mutations on ARID-DNA interactions requires the 3D structure of ARID-DNA complex, which is not available in the PDB and was generated *in silico*. For this purpose, we applied structural alignment between the KDM5C ARID domain (PDB ID: 2JRZ) and all available ARID-DNA complexes in PDB. The lowest RMSD value (2.22 Å) calculated from structural alignment (the alignment between the DNA binding interface of the KDM5C ARID domain and the ARID domain in the available complex structures) was found for the solution structure of the dead ringer ARID-DNA complex (PDB ID: 1KQQ) [41]. The dead ringer and the KDM5C ARID domains’ structural similarity (showed the lowest RMSD value (2.22 Å) calculated from structural alignment) was the highest for the residues situated at the protein-DNA interface, which suggested that the binding mode is preserved (Figure 9). Thus, the model ARID-DNA complex was built by superimposing the KDM5C ARID domain onto the dead ringer ARID-DNA complex and replacing the dead ringer ARID domain with the KDM5C ARID domain. Then, we saved the structure of the KDM5C ARID domain and DNA with untransformed coordinates as our model using the UCSF Chimera [30]. The DNA sequence in the model was kept the same as in the dead ringer ARID-DNA complex since the KDM5C ARID was not reported to show a DNA binding preference.

### 3.2. ARID Folding and Binding Free Energy Changes

We calculated the folding free energy change (∆∆G) and the binding free energy change (∆∆∆G) based on free energy perturbation theory (FEP) [42,43]. The free energy calculations of five mutations (A77T, D87G, R108W, N142S, and R179H) were performed with the NAMD program, version 2.9 [44] using alchemical transformations via the so-called dual topology approach [44,45], where both the initial and final states were defined concurrently. Periodic boundary conditions and a 12 Å cutoff distance for non-bonded interactions were applied in the system. Each FEP simulation was run using a CHARMM22 force field [46] and each mutation was carried out with one 18 ns run and four 5 ns runs. The initial protein structure used for each run was randomly taken from the trajectory of a 10 ns long equilibration. The results obtained with 18 ns and 5 ns runs were very similar and most of the 5 ns runs showed good convergence comparable with the convergence of 18 ns run (Supplementary Figure S1). This motivated us to carry the rest of the FEP using 5 ns simulations. Then, the output of FEP simulations was analyzed with the ParseFEP Plugin, Version 1.9 [47] in Visual Molecular Dynamics (VMD) [31]. Also, it has to be pointed out that Gly is a very particular case in FEP calculations since the library of hybrids contains the dual topologies for amino acids with a true side chain and the alpha carbon of Gly atom has to be modified in the transformation. For that reason, most patches cause problems and mutating glycine caused some angle and dihedral parameters to be duplicated, possibly modifying backbone conformational preferences [48]. Similar problems were also observed in our FEP calculation and, here, the FEP calculations of D87G were carried out for 1 ns with 0.5 fs time steps. For completeness, these calculated energies of D87G are used as they are in the present study.

**Figure 10:**
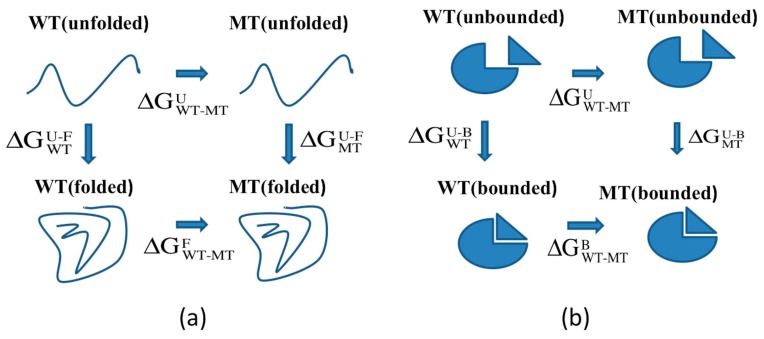
(**a**) Thermodynamic cycle for folding free energy changes calculations; and (**b**) thermodynamic cycle for binding free energy changes calculations.

The calculations of the effects of mutations on the folding free energy were performed utilizing the thermodynamic cycle we have developed in the past [49,50,51,52] (Figure 10a). The main assumption in this model is the unfolded state, which is considered to be made of two structural segments: (i) a structural three-residue segment centered at the mutation site; and (ii) the rest of the protein being mutation-independent [49,50,51]. This allows for canceling mutation-independent components of the unfolded state. Thus, the folding free energy change due to a mutation was calculated with the following equation:

(1) $$\left[\Delta \Delta G_{\text{folding}}=\Delta G_{\text{folding\_WT}}-\Delta G_{\text{folding\_MT}}=G_{\text{folded\_WT}}-G_{\text{unfolded\_WT}}^{\text{3}}-G_{\text{folded\_MT}}+G_{\text{unfolded\_MT}}^{3}\right]$$

where *G*^3^_unfolded_*X*_ is the free energy of the unfolded state of the three-residue segments at the center of mutation site, and *x* stands for WT or MT, respectively.

The effect of mutations on the binding free energy was calculated with the following thermodynamic cycle (see refs for more details [49,53,54,55,56]) (Figure 10b), and the corresponding equation is provided below:

(2) $$\left[\Delta \Delta G_{\text{binding}}=\Delta G_{\text{binding\_WT}}-\Delta G_{\text{binding\_MT}}=G_{\text{bounded\_WT}}-G_{\text{unbounded\_WT}}-G_{\text{bounded\_MT}}+G_{\text{unbounded\_MT}}\right]$$

where the unbounded state means the protein is taken away from its partner and bounded state means the protein forms a complex with its partner protein.

### 3.3. Utilizing Webservers and Third Party Software

Third-party methods were also used to predict protein folding free energy change, including webservers and stand-alone computer algorithms. The webservers used to predict the folding free energy changes upon single point mutations include NeEMO [57], PopMusic [58], I-Mutant 2.0 [59], DUET [60], and CUPSAT [61]. Additionally, a computer algorithm, FoldX 3.0 Beta3 [62,63], was used to predict the folding free energy changes upon single-point mutations. Currently, no reliable third-party software or a functioning webservers for predicting binding free energy changes are available.

### 3.4. Molecular Dynamics Simulation

We carried out MD simulations to investigate mutations’ effects on the dynamics on the ARID domain. The simulations were set up within the NAMD program, version 2.9 [44], using the CHARMM22 force field [46]. The PDB structure taken from Protein Data Bank [22] was used as the initial structure. To relax conflicting contacts, energy minimization was performed using the conjugate gradient energy minimization of 10,000 steps. The protein was solvated in a water box with a layer of water extending 10 Å in each direction before the minimization and equilibration with periodic boundary conditions. Temperature and pressure in the simulation were set to 298 K and 1 bar. Each mutation was repeated for three 100 ns runs using 2 fs time steps. The trajectory files were analyzed by using VMD plugins [31] in order to obtain the RMSD, RMSF, and salt bridges.

### 3.5. Electrostatic Potential Calculation

The DelPhi program was used to perform the electrostatic potential calculations using the following parameters: scale = 2 grid/Å; percentage of protein filling of the cube = 70%; dielectric constant = 2 for the protein and 80 for the solvent; and water probe radius = 1.4 Å. We outputted the DelPhi-calculated potential map into a file in CUBE format, which was further opened and analyzed in UCSF Chimera.

### 3.6. ARID Domain Protein Expression and Purification 

The ARID (residues 73–188) of KDM5C was amplified from plasmid containing KDM5C cDNA (pENTR221) available from DNASU [64] by PCR using forward primer (5’-AATCCAGCATATGAATGAGCTAGAGGCCCAG-3’) and reverse primer (5’-GATAATGAGGAGAAGGACAAGTAAAAGCTTAATATT-3’), which contained NdeI and HindIII restriction sites (underlined), respectively. The amplified DNA fragments and pET28b vector were digested with NdeI and HindIII, ligated, and transformed into *E. coli* strain DH5α chemically-competent cells. The ARID wild-type (WT) plasmid was sequenced and then used as the template to generate the A77T and D87G variants by using the overlapping primer extension methods. Listed below are the primers (purchased from Eurofins MWG Operon LLC, Huntsville, AL, USA) for A77T and D87G variant generation:

ARID A77T NdeI F1 (5’-CATATGAATGAGCTAGAGACCCAGACGAGAGTGAAAC-3’)
ARID A77T NdeI R2 (5’-GTTTCACTCTCGTCTGGGTCTCTAGCTCATTCATATG-3’)
ARID D87G F1 (5’-GAAACTGAACTACTTGGGCCAGATTGCCAAATTCTG-3’)
ARID D87G R2 (5’-CAGAATTTGGCAATCTGGCCCAAGTAGTTCAGTTTC-3’)

The PCR condition for generating the wild-type ARID is as follows: 50 ng DNA template (pENTR221), 0.2 mM dNTPs mix (Promega, Fitchburg, WI, USA), 0.4 µM primers (Forward and Reverse), and 1 U of Pfu DNA polymerase. The reactions were carried out in 50 µL of 1× Pfu buffer (20 mM Tris∙HCL (pH 8.8), 10 mM KCl, 10 mM (NH_4_)_2_SO_4_, 2 mM MgCl_2_, 0.1% Triton X-100, 0.1 mg/mL nuclease-free BSA). The reactions were performed using Mastercycler Gradient (Eppendorf, Hauppauge, NY, USA) with the following steps:

1. Initial denaturing at 95 °C for 1:30 min
2. 95 °C for 30 s
3. Annealing at 60 °C for 35 s
4. Extension at 72 °C for 1 min

Steps 2–4 were done for 30 cycles, followed by a final extension for 10 min at 72 °C and then the reaction was cooled down to 4 °C.

Mutants’ ARID genes were generated using the same condition as the wild-type except the DNA template was the ARID WT in pET28b. The PCR to create the inserts for the mutants are done in two steps, the first step is to generate two PCR fragments using the forward primer with T7 Terminator (PCR1) and reverse primer with T7 Promoter (PCR2). The two PCR fragments were purified by gel extraction and used as the template for the second round of PCR with T7 Promoter and T7 Terminator primers to generate the complete the mutants’ ARID genes. The amino acids sequence of the recombinant ARID wild-type is:

*MGSSHHHHHHSSGLVPRGSHM*NELEAQTRVKLNYLDQIAKFWEIQGSSLKIPNVERRILDLYSLSKIVVEEGGYEAICKDRRWARVAQRLNYPPGKNIGSLLRSHYERIVYPYEMYQSGANLVQCNTRPFDNEEKDK

The two amino acids subjected to mutation (Ala77 and Asp87) are underlined and the italicized residues are the His_6_-tag and linker from the expression system. 

The vectors carrying the WT, A77T, and D87G were confirmed by DNA sequencing and transformed into *E. coli* Rosetta™ 2(DE3) pLysS (Novagen, Cambridge, MA, USA). The protein overexpression was done by following the standard protocol from the manufacturer. Briefly, a single colony carrying the plasmid which contained the protein of interest was grown overnight in 5 mL culture of LB and 50 µg/mL kanamycin at 37 °C. The overnight culture was diluted 500-fold into 1 L LB containing 50 µg/mL kanamycin, grown in a shaker incubator at 37 °C with 250 rpm shaker speed, when the OD_600_ reached 0.6, isopropyl-1-thio-α-*d*-galactopyranoside (IPTG) was added to a final concentration of 1 mM and the culture was left to grow for 3 h. The cells were harvested by centrifugation at 5000× *g* for 15 min at 4 °C. The cell pellet was washed once with pre-cooled Buffer A (20 mM Tris-HCl pH 7.5, 150 mM NaCl and 20 mM imidazole, 1 mM PMSF) and then re-suspended in 25 mL Buffer A and sonicated at output 5 for 3 × 1 min with 5 min rest on ice between intervals using Qsonica model Q125. The crude lysate was clarified by centrifugation at 20,000× *g* for 10 min twice. The supernatant was filtered through a 0.45 micron syringe filter before it was applied onto 5 mL HisTrap FF column (GE-Healthcare, Huntsville, AL, USA). The bound proteins were eluted with a linear gradient of 0%–100% Buffer B (20 mM Tris-HCl pH 7.5, 150 mM NaCl and 500 mM imidazole). Fractions containing the ARID protein were identified by SDS-PAGE, pooled, diluted eight-fold with buffer QA (20 mM Tris-HCl pH 8) and applied onto a 5 mL HiTrap Q FF column preequlibrated with Buffer QA. The ARID protein was purified by linear gradient elution (50–1000 mM NaCl). The eluted ARID protein was concentrated and exchanged to a storage buffer (20 mM Tris-HCl (pH 7.5), 1 mM DTT, 150 mM NaCl, and 40% Glycerol) by using Microcon YM 10 (Millipore, Billerica, MA, USA) spin column. Protein concentration was determined by UV spectroscopy (BioTek™ Eon™ Microplate Spectrophotometers, BioTek, Winooski, VT, USA) using a calculated extinction coefficient (ε) of 22,920 M^−1^·cm^−1^ at 280 nm [65].

### 3.7. Circular Dichroism Spectrum and Urea-Induced Unfolding

Circular dichroism (CD) spectra of ARID proteins (20–30 µM) at 25 °C were measured in a quartz cuvette (Starna Cells, Atascadero, CA, USA) with 0.1 cm path length using a JASCO J-810 spectropolarimeter (ASCO Inc., Easton, MD, USA) at different concentrations of urea to induce ARID unfolding [66]. For each CD spectrum, ellipticity and absorbance values were obtained over a wavelength from 220 to 300 nm, at a scan rate of 100 nm/min and a response time of 0.25 s. Six scans were performed per protein at each urea concentration of urea and the CD value were averaged. The difference between the absorbance at 280 nm and 300 nm was used for calibrating protein concentration. The mean residue ellipticity ([θ]) (degree·cm^2^·dmol^−1^·residue^−1^) was converted from corrected CD signals ${\text{θ}}_{\text{λ}}$ by Equation (3) [66]:

(3) $$\left[\left[\text{θ}\right]=\frac{{\text{θ}}_{\text{λ}}}{10\;\times \;l\;\times \;C\;\times \;N}\right]$$

where *l* is the path length of the cuvette in cm, *C* is the protein concentration in molar, and *N* is the number of amino acid residues. The fraction of denatured protein ($f_{d}$) at a certain urea concentration and the apparent free energy of denaturation ($\Delta G_{\text{app}}$) were calculated from the mean residue ellipticity at 222 nm (${\left[\text{θ}\right]}_{222}$) using Equations (4) and (5) by assuming a two-state transition model [67]:

(4) $$\left[f_{d}=\frac{{\left[\text{θ}\right]}_{222}-{\left[\text{θ}\right]}_{222}^{N}}{{\left[\text{θ}\right]}_{222}^{D}-{\left[\text{θ}\right]}_{222}^{N}}\right]$$

(5) $$\left[\Delta G_{\text{app}}=-RTln\left(\frac{f_{d}}{1-f_{d}}\right)\right]$$

where ${\left[\text{θ}\right]}_{222}^{N}$ is the mean residue ellipticity at 222 nm of protein in the native state $\;{\left[\text{θ}\right]}_{222}^{D}$ is the mean residue ellipticity of fully-denatured protein, *R* is the ideal gas constant, and *T* is the absolute temperature. Free energy of denaturation in H_2_O ($\Delta G_{\text{app}}^{{\text{H}}_{2}\text{O}}$) can be obtained with Equation (6) by fitting a straight line through the plot of free energy *versus* urea concentration:

(6) $$\left[\Delta G_{\text{app}}^{\left[Urea\right]}=\;\Delta G_{\text{app}}^{{\text{H}}_{2}\text{O}}-m\left[Urea\right]\right]$$

where [*Urea*] is urea concentration and *m* is the slope of the fitted line. The difference of the apparent free-energy of denaturation of the ARID wild-type and mutants are calculated using Equation (7) [68,69] or Equation (8) [70,71]:

(7) $$\left[\Delta \Delta G_{\text{app},1}^{{\text{H}}_{2}\text{O}}=\;\Delta G_{\text{app},\text{wildtype}}^{{\text{H}}_{2}\text{O}}-\Delta G_{\text{app},\text{mutant}}^{{\text{H}}_{2}\text{O}}\right]$$

(8) $$\left[\Delta \Delta G_{\text{app},2}^{{\text{H}}_{2}\text{O}}=\;\langle m\rangle \Delta C_{\text{m}}\right]$$

where 〈m〉 is the average of the slopes of all urea denaturation curves and $\Delta C_{\text{m}}$ is the difference between the ARID wild-type and mutant protein denaturant midpoint ([*Urea*]_1/2_).

## 4. Discussion and Conclusions

The KDM5C ARID domain binds to DNA and the formation of an ARID-DNA complex is important for the KDM5C function in humans [10,12,35]. Our analysis shows that A77T and D87G have minimal effect on the ARID domain’s DNA binding, which indicates that the disease-associated mechanism is probably not due to the alteration of DNA binding. It is also interesting that both of the disease-associated mutations are located onto the N-terminal of the ARID domain and both of the mutations are far away from the ARID domain’s DNA binding interface. We speculate that some not-yet-discovered function of the KDM5C protein is associated with the ARID domain’s N-terminal. To test this, we analyzed the KDM5C ARID domain using the ConSurf Server [72,73,74,75]. The Consurf results support our speculation and show that both of the disease-associated mutations are located in the most highly-conserved part of the ARID domain and possibly cause a change in an important function of the protein. Additionally, D87 is predicted to be a plausible zinc ion binding site and further supports that some currently-unknown function is linked to N-terminal of the ARID domain.

Previous studies show that KDM5C is a muti-functional protein and inter-domain interactions are identified among the JmjN domain, N-PHD domain, and JmjC domain [7,12,19]. The interaction between the JmjC domain is important for the demethelytion activity. The N-PHD domain and JmjC domain can bind to the same histone tail at Lys4 and Lys9. Both of pathogenic mutations happen in the N-terminal of the ARID domain and are close to the linker part between the ARID and JmjN domains. This suggests that A77T and D87G may be involved in some unknown interaction among JmjN, ARID, PHD, and JmjC domains. Currently, only the ARID domain structure is available and the arrangement of the KDM5C domain is unknown.

Our study also evaluates three non-classified mutations’ effects on the KDM5C ARID domain. Among them, the R108W causes a loss of a salt bridge, slightly affecting protein’s stability and ARID-DNA binding affinity. Therefore, we speculate that R108W is a disease-associated mutation based on altering structural features rather than on the calculated free energy changes. In addition, as demonstrated, R108W changes the electrostatic potential near the DNA binding site which may affect the specificity of ARID-DNA binding. 

In our work, protein binding and folding energy changes were calculated with FEP, webservers, and third party software. Limitation about the technical issues in FEP calculations are observed for the Arg- and Gly-involved mutations, possibly causing less reliable predictions. Therefore, other methods, including webservers and third-party software were also applied in the free energy calculation to compare with the FEP results. Furthermore, the experimental results of the mutants A77T and D87G are obtained by urea-induced unfolding methods, showing the same order of magnitude compared to the folding free energy calculation. Another limitation about this work is that our speculation about the unknown function in the N-terminal of the ARID domain has not been experimentally verified and, currently, the only known function about the ARID domain is the DNA binding interaction. However, our work implicates that the sites 77 and 87 may be involved in some other function or interaction different from cognate ARID-DNA binding. This provides motivation for future studies to further investigating other functions of KDM5C.

## Supplementary Materials

Supplementary materials can be found at http://www.mdpi.com/1422-0067/16/11/26022/s1.
