# Supplementary material for: Mutations in the KDM5C ARID Domain and Their Plausible Association with Syndromic Claes-Jensen-Type Disease
Source: Int J Mol Sci. 2015 Nov 13;16(11):27270–87. doi: 10.3390/ijms161126022 (PMC4661880; doi:10.3390/ijms161126022)
Supplement: Supplementary file 1 [file ijms-16-26022-s001.pdf]

Supplementary Materials: Mutations in the KDM5C ARID Domain and Their Plausible Association with Syndromic Claes-Jensen-Type Disease

Yunhui Peng, Jimmy Suryadi, Ye Yang, Tugba G. Kucukkal, Weiguo Cao and Emil Alexov

In the study, the FEP calculation of each mutation includes one 18 ns run and four 5 ns runs. To compare the accuracy, we plot the evolution of the free energy as a function of the number of molecular-dynamics steps for both the forward and the backward transformations. It is showed that most of the 5 ns runs show good convergence comparable with the convergence of 18 ns run. Supplementary Figure S1 takes evolution of the free energy of A77T as an example to compare the convergence between 18 ns and 5 ns.

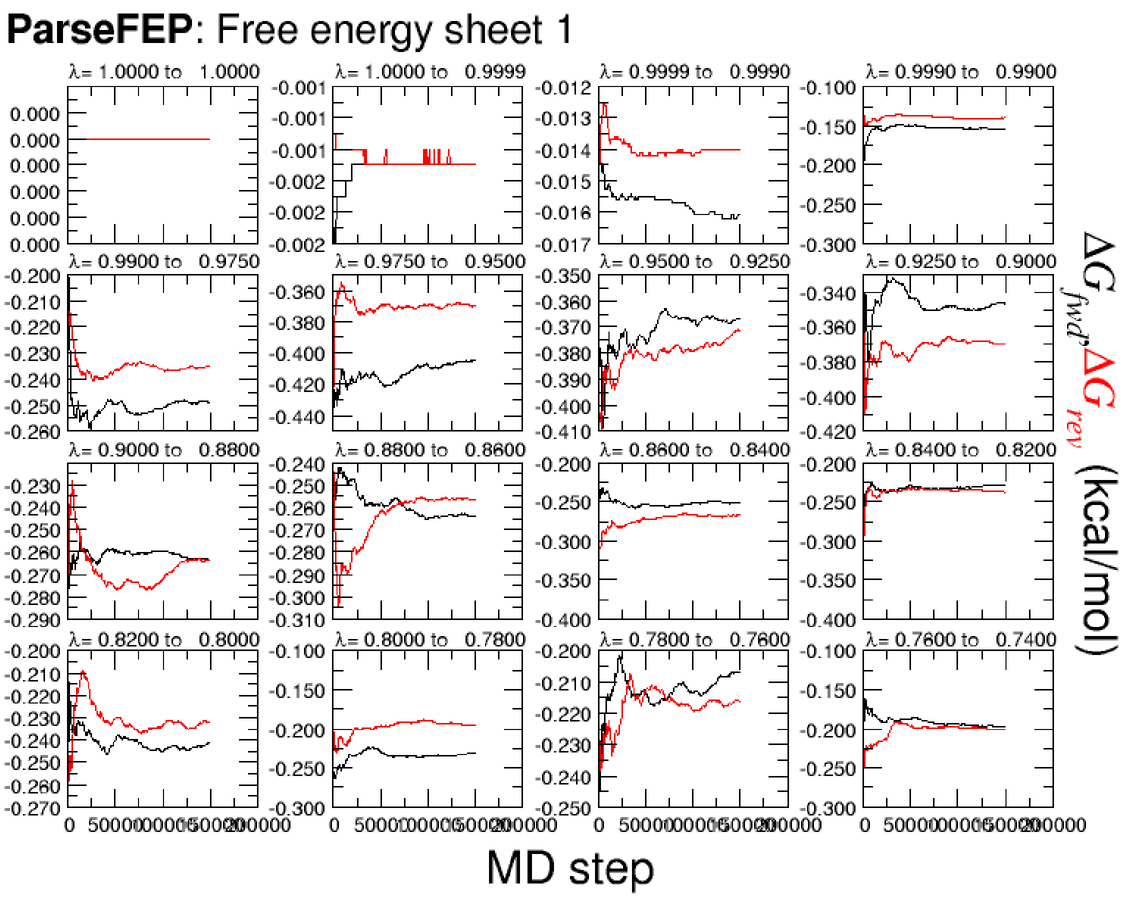

(a)

Figure S1. *Cont.*

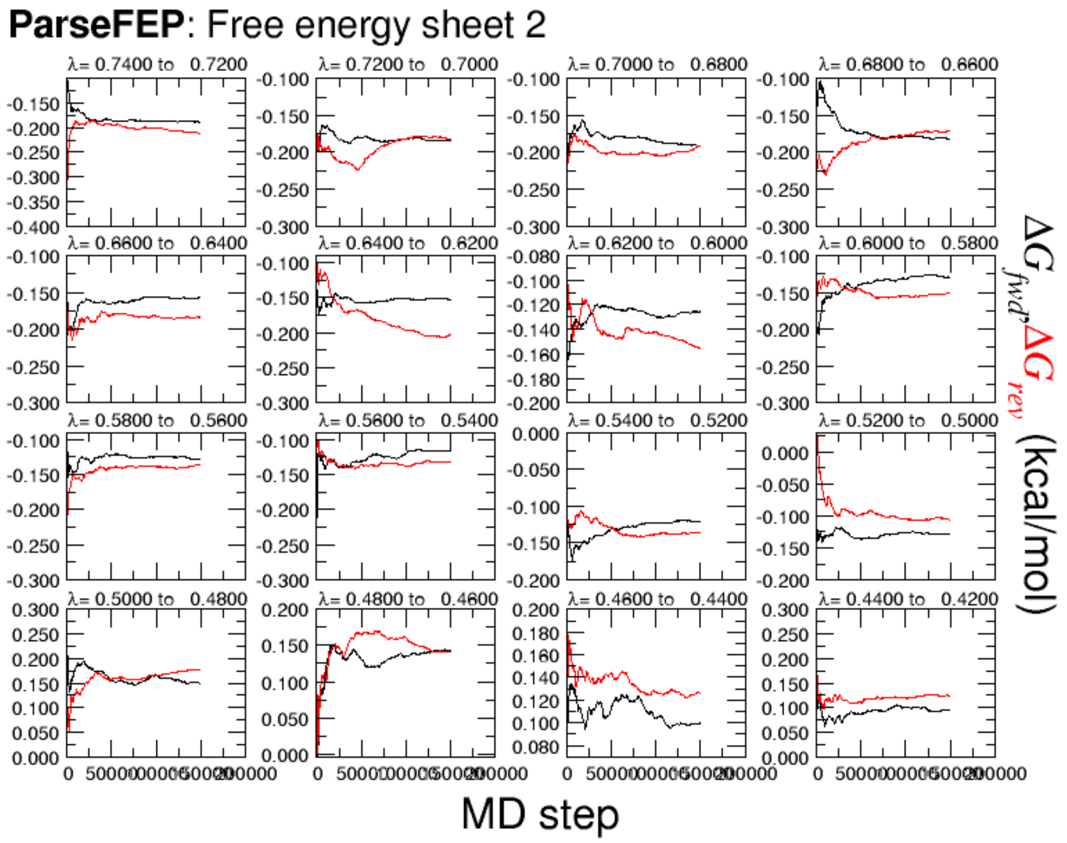

(**b**)

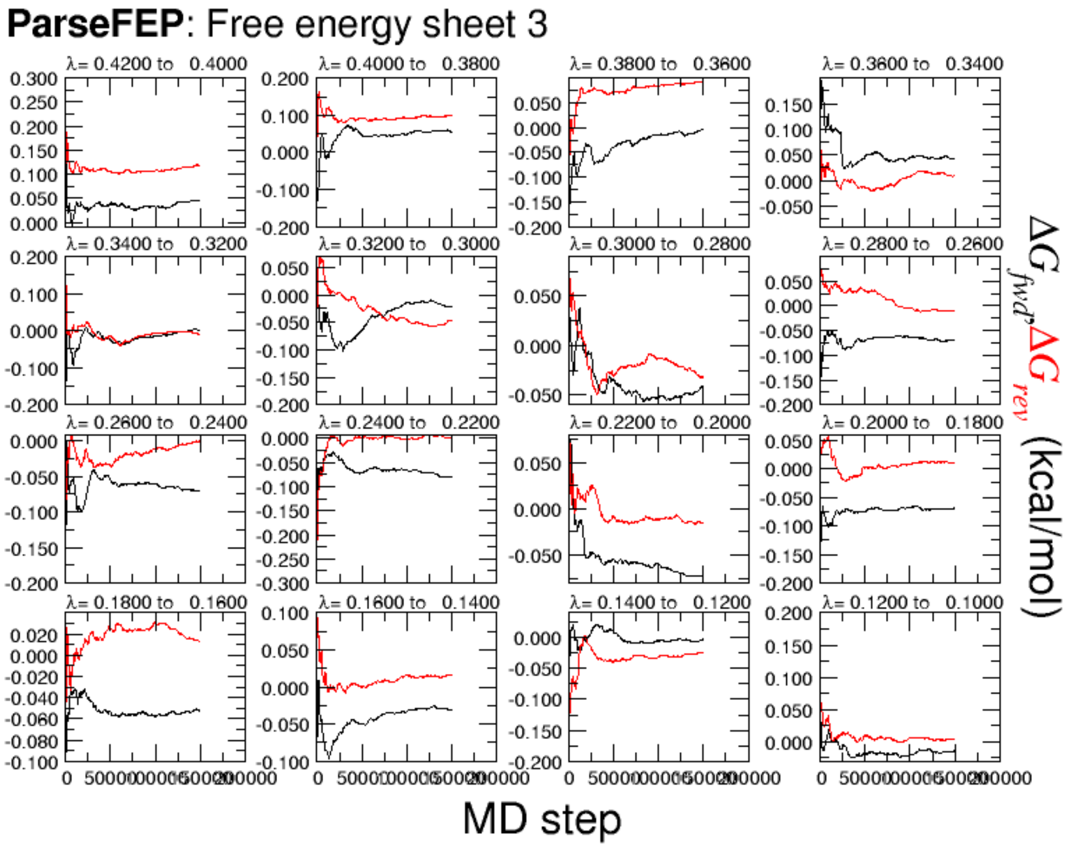

(**c**)

Figure S1. *Cont.*

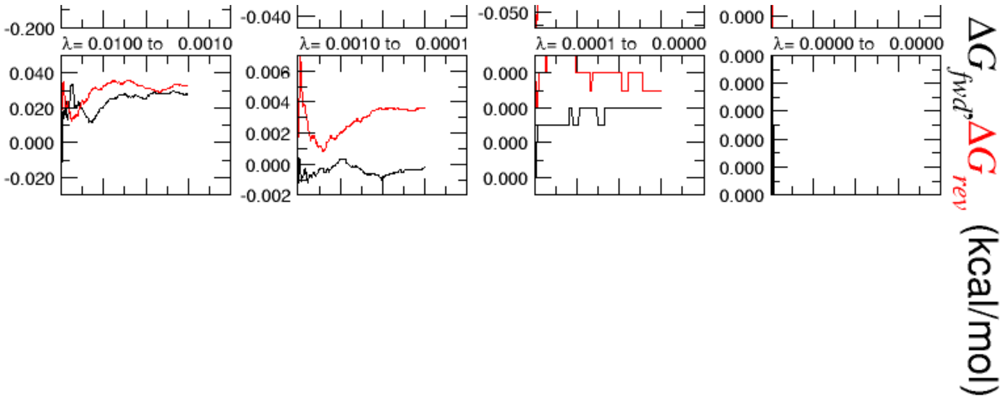

(**d**)

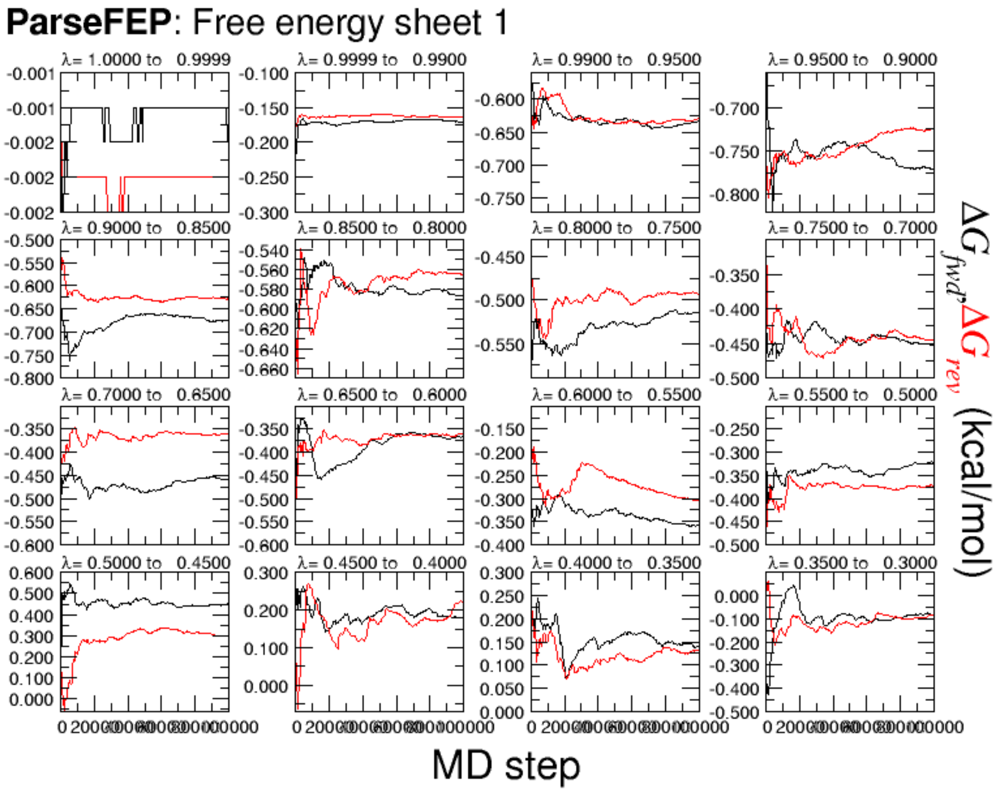

(**e**)

Figure S1. *Cont.*

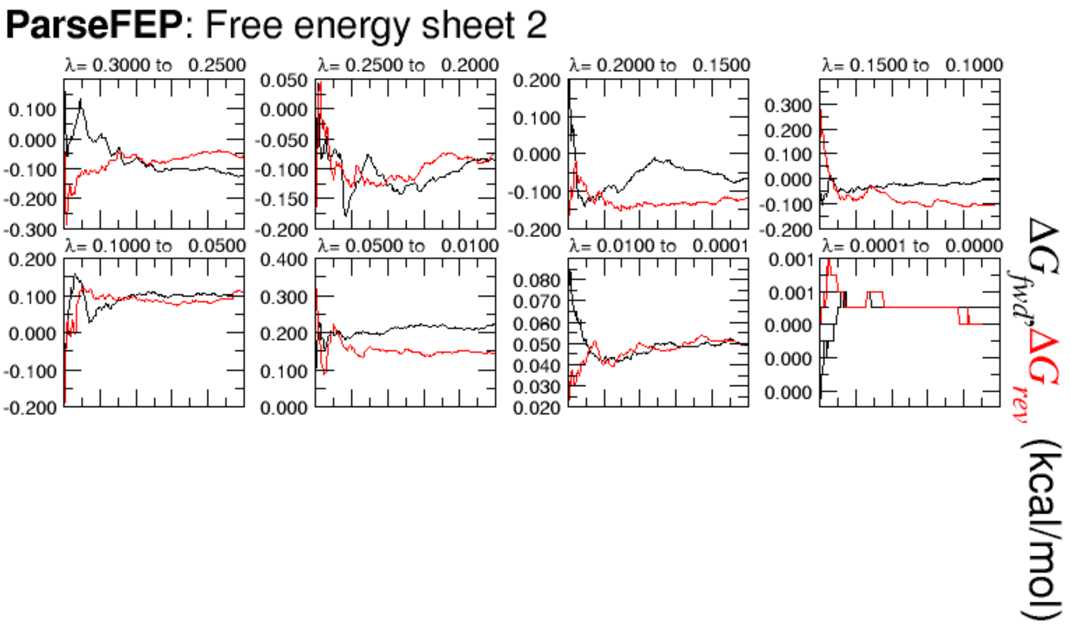

(f)

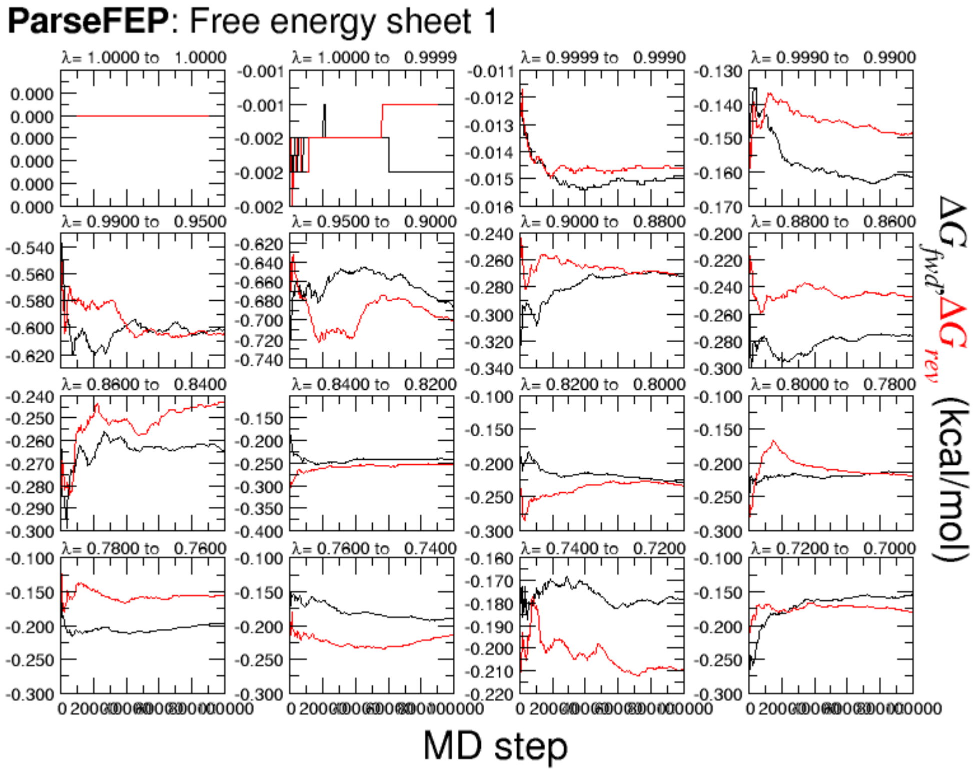

(g)

Figure S1. *Cont.*

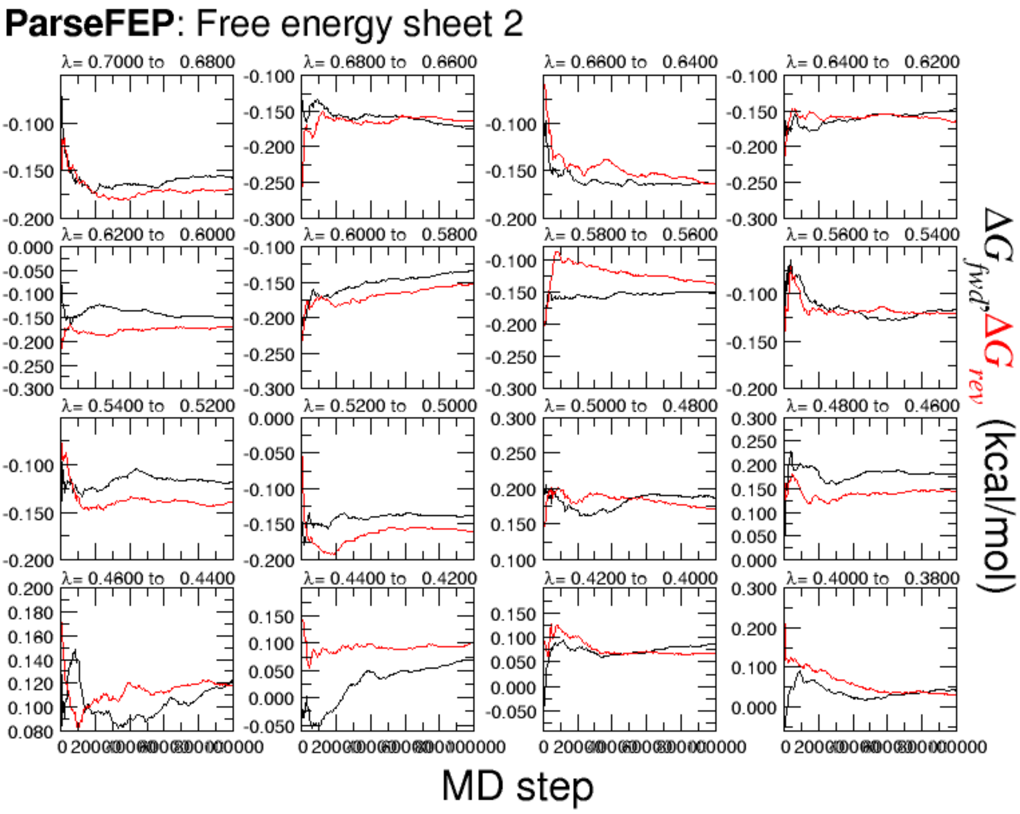

(**h**)

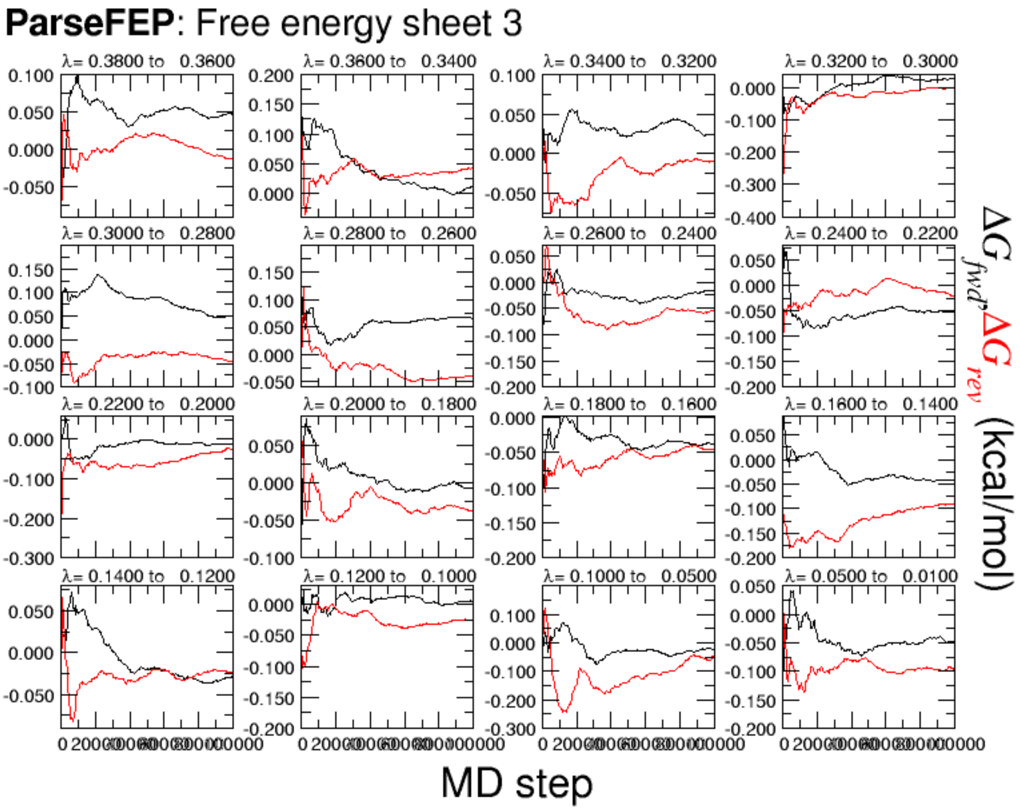

(**i**)

Figure S1. *Cont.*

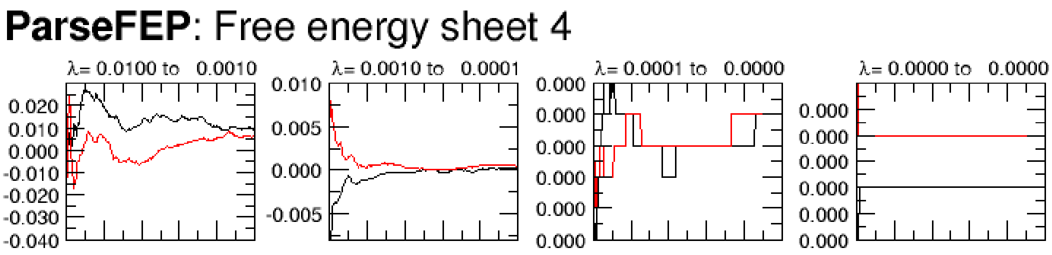

(j)

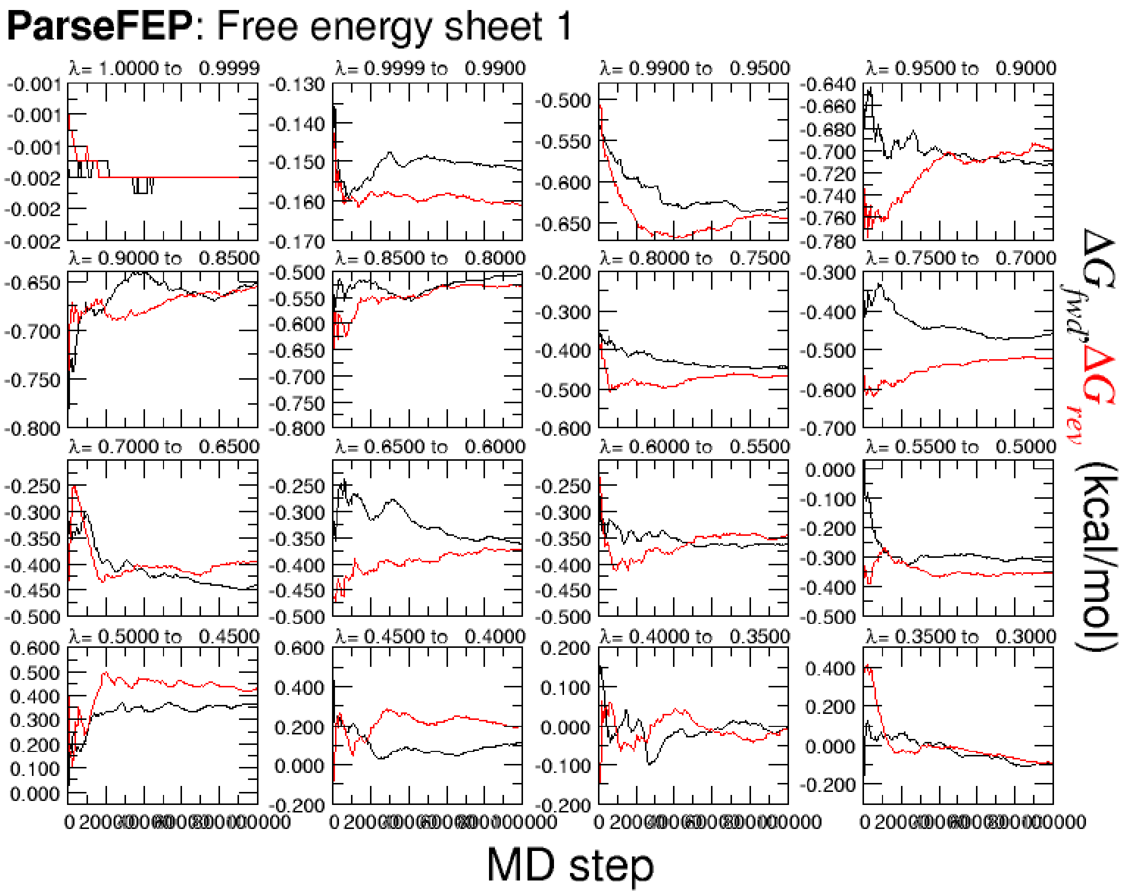

(k)

Figure S1. *Cont.*

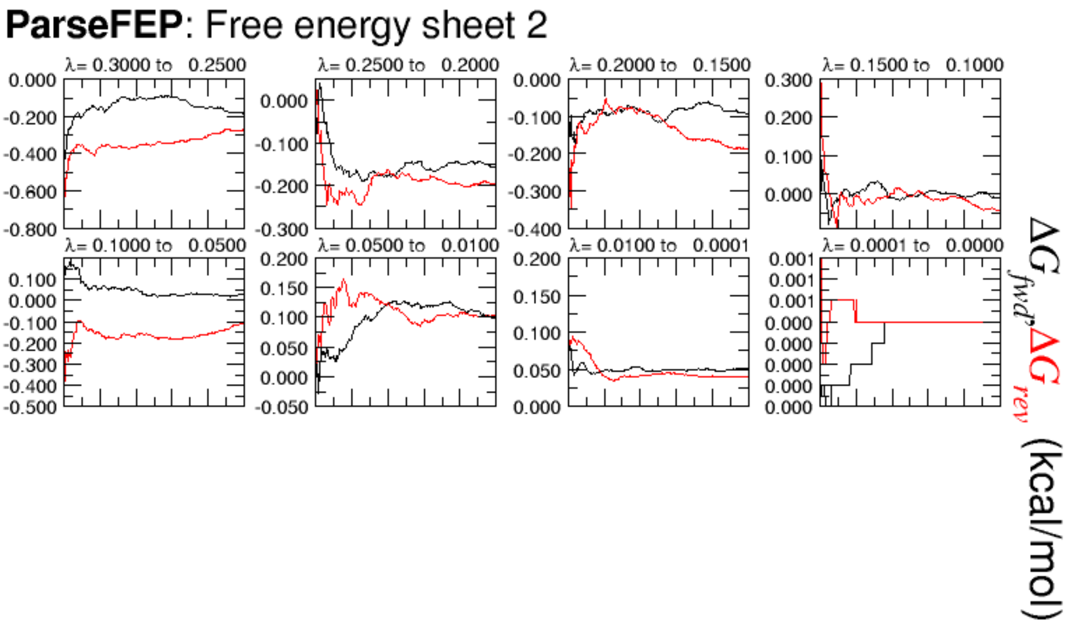

(l)

Figure S1. Evolution of the free energy for A77T binding free energy calculation and folding free energy calculation. (**a**–**d**) show evolution of the free energy as a function of the number of molecular-dynamics steps in 18ns run for binding free energy calculation. (**e**,**f**) show evolution of the free energy as a function of the number of molecular-dynamics steps in 5ns run for binding free energy calculation. (**g**–**j**) show evolution of the free energy as a function of the number of molecular-dynamics steps in 18ns run for folding free energy calculation. (**k**,**l**) show evolution of the free energy as a function of the number of molecular-dynamics steps in 5 ns run for folding free energy calculation.

Supplementary Figure S2 shows the RMSD calculations of ARID domain and the mutation occurring local regions for mutations A77T, D87G, R108W, N142S and R179H.One of run for D87G mutant shows relative large RMSD value comparing to other five runs, which probably comes from the error in the sampling. D87G also show minimal effect on the RMSD value of helix1, which indicates that the mutation probably doesn't affect the backbone of the helix. The analysis also shows that the RMSD value of A77T mutation occurring local regions varies between 1.5 Å and 4 Å for WT and MT. This is probably due to that A77T is located onto a loop part and has very high flexibility in the simulation. For non-classified mutations, we do not observe clear tendency that the mutations tend to decrease or increase the RMSD value of the whole ARID domain or the mutation happening part.

Also, we studied the mutations' effect on the protein flexibility by calculating Root Mean Square Flexibility (RMSF). Supplementary Figure S3 shows the flexibility of each residue in the 100 ns simulation from 5 runs. D87G is located onto the helix part and the RMSF doesn't show obvious tendency that the mutations increase the flexibility of helix1. Other mutations all are located onto the loop part and shows a relative high flexibility in our analysis. RMSF also does not shows obvious tendency to increase or decrease comparing the WT and MT.

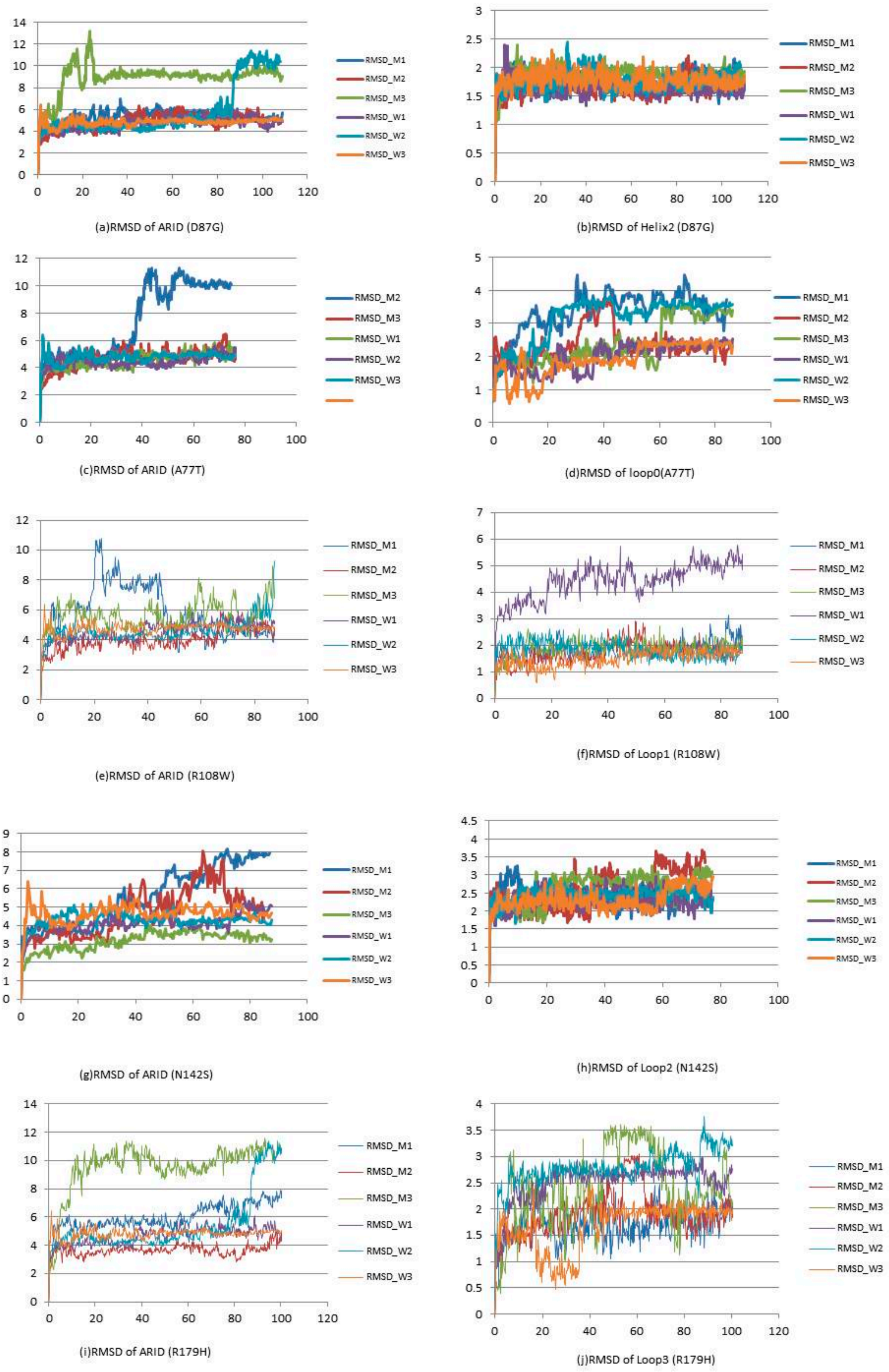

Figure S2. RMSD calculation. (**a**,**c**,**e**,**g**,**i**) shows the RMSD calculations of ARID domain for mutations A77T, D87G, R108W, N142S and R179H; (**b**,**d**,**f**,**h**,**j**) shows the local RMSD calculation for the mutations, where the RMSD is only calculated within the mutation occurring local regions. Helix2 includes residue 82–95. Loop0 includes residue 5–12. Loop1 includes residue 96–112. Loop2 includes residue 140–148. Loop3 includes residue 173–181.

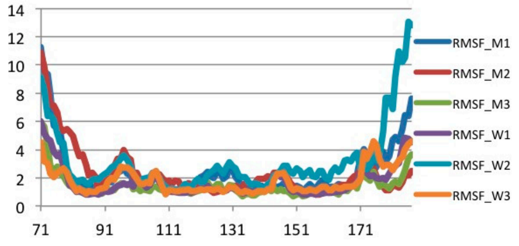

(a)RMSF of ARID(A77T)

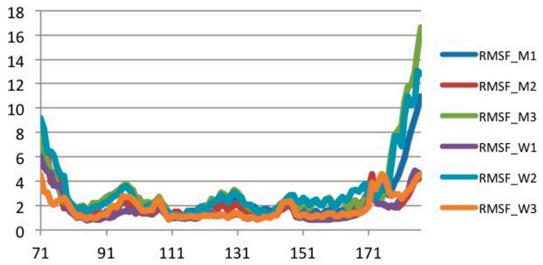

(b)RMSF of ARID(D87G)

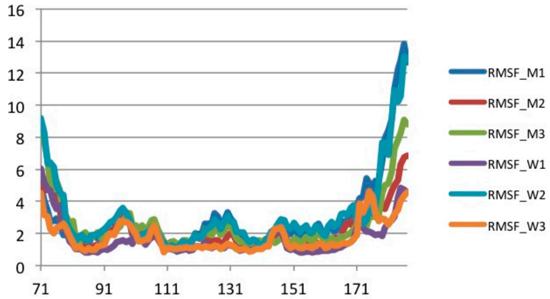

(c)RMSF of ARID(R108W)

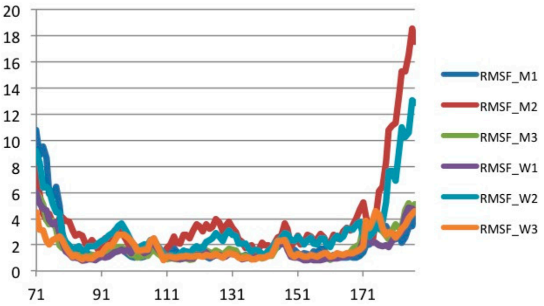

(d)RMSF of ARID(N142S)

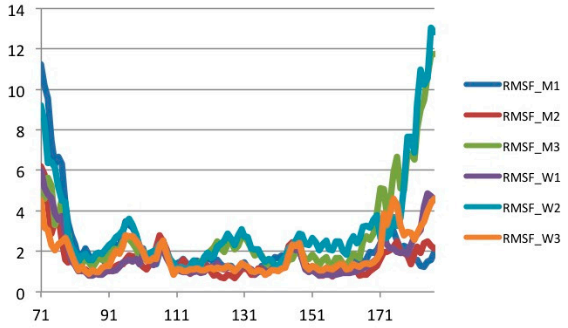

(e)RMSF of ARID(R179H)

Figure S3. RMSF calculation. (**a**–**e**) show the RMSF calculations of ARID domain for mutations A77T, D87G, R108W, N142S and R179H.
